# Review of Copper and Copper Nanoparticle Toxicity in Fish

**DOI:** 10.3390/nano10061126

**Published:** 2020-06-07

**Authors:** Nemi Malhotra, Tzong-Rong Ger, Boontida Uapipatanakul, Jong-Chin Huang, Kelvin H.-C. Chen, Chung-Der Hsiao

**Affiliations:** 1 Department of Biomedical Engineering, Chung Yuan Christian University, Chung-Li 32023, Taiwan; nemi.malhotra@gmail.com (N.M.); sunbow@cycu.edu.tw (T.-R.G.); 2 Department of Applied Chemistry, National Pingtung University, Pingtung 90003, Taiwan; hjc@mail.nptu.edu.tw; 3 Department of Applied Chemistry, Faculty of Science and Technology, Rajamangala University of Technology Thanyaburi, Thanyaburi 12110, Thailand; boontida_u@rmutt.ac.th; 4 Department of Chemistry, Chung Yuan Christian University, Chung-Li 32023, Taiwan; 5 Department of Bioscience Technology, Chung Yuan Christian University, Chung-Li 32023, Taiwan; 6 Center for Nanotechnology, Chung Yuan Christian University, Chung-Li 32023, Taiwan

**Keywords:** copper, copper nanoparticle, toxicity, fish

## Abstract

This review summarizes the present knowledge on the toxicity of copper and copper nanoparticles (CuNPs) to various fish species. In previous decades, the excessive usage of metal and metallic nanoparticles has increased significantly, increasing the probability of the accumulation and discharge of metals in various trophic levels of the environment. Due to these concerns, it is important to understand the toxicity mechanisms of metals and metallic nanoparticles before they lead to unhealthy effects on human health. In this review paper, we specifically focus on the effect of metal copper and CuNPs on different fish organs under different physiochemical parameters of various water bodies. Nowadays, different forms of copper have distinctive and specific usages, e.g., copper sulfate is a well-established pesticide which is used to control the growth of algae in lakes and ponds. Deactivating the fungi enzymes prevents fungal spores from germinating. This process of deactivation is achieved via the free cupric ions, which are established as the most toxic forms of copper. Complexes of copper with other ligands may or may not be bioavailable for use in aquatic organisms. On the other hand, CuNPs have shown cost-effectiveness and numerous promising uses, but the toxicity and availability of copper in a nanoparticle form is largely unknown, Additionally, physiochemical factors such as the hardness of the water, alkalinity, presence of inorganic and organic ligands, levels of pH, and temperature in various different water bodies affect the toxicity caused by copper and CuNPs. However, comprehensive knowledge and data regarding the pattern of toxicity for copper metal ions and CuNPs in marine organisms is still limited. In this review, we carry out a critical analysis of the availability of the toxicological profiles of copper metal ions and CuNPs for different fishes in order to understand the toxicity mechanisms of copper and CuNPs. We believe that this review will provide valuable information on the toxicological profile of copper, which will further help in devising safe guidelines for the usage of copper and CuNPs in a sustainable manner.

## 1. Introduction

In recent years, copper and copper-based nanoparticles (CuNPs) have been used for industrial purposes [1,2], electrical equipment [3], construction materials [4], antimicrobial agents [5], and alloy formation with other metals. CuNPs are increasingly used in various sectors, including as catalysts in organic synthesis [6,7,8], for drug delivery [9], sensors [10,11,12,13,14], agriculture and food preservation [15,16,17,18], and paint and water treatment [19,20]. There is an abundant supply of copper in the earth’s crust [21]. Copper is a ductile and malleable heavy metal with a density greater than 5 g/cm^−3^ and low chemical reactivity. Copper is also an essential trace micronutrient that plays a significant role as a co-factor in critical enzyme reactions related to body processes necessary for survival in both human and animals [22,23,24]. In addition to the wide variety of uses of copper, it is also involved in enzymatic activities, including lysyl oxidase, tyrosinase, and dopamine hydroxylase. It is associated in its metabolic roles with the formation of copper chelates and complexes of Cu proteins [25]. Copper plays an important biological role in oxygen transportation as part of hemocyanin. Hemocyanin is a counterpart of hemoglobin for oxygen transportation, which is found in mollusks and crustaceans [26].

Hence, to understand the mechanism of copper toxicity to organisms, first it is essential to understand its dominance as a chemical and its behavior in the environment [27]. Metals react based on their soluble properties in an aquatic medium. The free ions or complexes generated by metals can be absorbed on suspended particulates in the aquatic medium [28]. Metal constituents might behave differently in an aqueous system. With respect to the earlier statement, copper levels in waterbodies must always be maintained at low levels. Unpolluted water has a copper level as low as 0.5 to 1 µg/L (ppb) [29].

In water bodies, the speciation of copper strongly affects the ability of copper to create toxicity [30,31]. Copper is a transition metal with three oxidation states, namely Cu(0) (solid metal state), Cu(I) (cuprous ion), and Cu(II) (cupric ion). Copper is an essential bioactive trace metal in marine environments and an important micronutrient for many aquatic species [32]. The oxidation of Cu(I) to Cu(II) provides the blue tinge in mollusks and crustaceans due to the presence of hemocyanin protein. In complex forms, copper is less bioavailable and less toxic than the free ionic form Cu^2+^ [32,33,34,35]. The different oxidation states of copper are used to design nanoparticles with various sets of distinctive properties. The highly conductive elemental copper (Cu(O) or nCu) can trigger electron transfers [36,37]. Cu(I), used in Cu_2_O NPs, can flip between Cu^+^ and Cu^2+^, whereas Cu(II) can be synthesized in the form of Cu(OH)_2_ NPs and can be used as an antimicrobial agent. Accordingly, it becomes necessary to evaluate the release of copper ion forms in the marine environment in order to analyze the toxicity and bioavailability if accumulated in an environment containing aquatic biota.

The similarities of copper metabolism in fish and mammals were studied by Syed and Coombs [38]. Most of the copper was found in the gills, kidney, brain, liver, and skeletal muscle of the fish [39]. Copper is an important compound for the maintenance of red blood cells, nerve cells, and the immune system. With an improper metabolism of copper, accumulation of this element may be harmful in humans. Copper imbalance in the body has been linked to certain genetic diseases, such as Menkes disease and Alzheimer’s disease [40]. In 2001, a recommended dietary allowance of copper was introduced as 0.9 mg/d for adults [41,42]. However, studies have shown that a high concentration of copper is toxic in both fish and humans [43].

A significant amount of copper is usually found near copper mines [44]. Aquatic habitats such as lakes, rivers, and oceans are most vulnerable to any kind of metal pollution, because all of the industrial waste, weathering of soil, and urban mining is discharged into the water bodies, which in turn affects the aquatic biota. The monitoring of aquatic ecosystems is essential because they support a wide range of organisms, including microorganisms, plants, insects, and fish, thus maintaining healthy biodiversity. Heavy metals do not degrade; instead, they are assimilated or absorbed in water sediment and aquatic animals, causing metal pollution in their bodies [45].

This absorption of metal affects aquatic organisms directly, for instance via an increase in solubilization and mobilization, which is related to modification of their bodies. Multiple factors such as alkalinity, hardness, redox potential, and the organic and oxygen contents of water have been known to affect metal accumulation inside aquatic organisms [46,47,48,49,50]. The form of the metal (ionic, complexed, and precipitated), in association with the physiochemical factors of varying environments, affects the bioavailability of the metal to the aquatic biota, giving rise to conditions of metal deficiency or toxicity [27].

The assessment of copper levels in the ground, as well as marine environments around the world, involves comprehensive analysis of surface water and sediment [21,51]. Moreover, the sensitivity of fish and other aquatic organisms to dissolved metals or other impurities depends on their surface area to volume, flow rates over gill surfaces, and respiratory rates. The modification of these parameters allows the tolerance of copper metal uptake to be measured by monitoring the increase or decrease in copper uptake and the effects thereof [27].

Fishes and shellfish are important parts of the diets of seafood-dependent countries [52]. Correspondingly, fishes and shellfish are well-known indicators of heavy metal pollution [53]. Examining the metal concentration in fish and shellfish meat is especially important to ensure compliance with food safety regulations and consumer protection, because once metal assimilates in biota, it tends to biomagnify in the food chain and becomes difficult to break down into less dangerous compounds.

Toxicological tolerance limits in mammals are generally 10- to 100-fold higher than those of fish or crustaceans [21]. To further analyze aquatic toxicology, a biotic ligand model (BLM) was developed to explain and predict the effects of metal toxicity to aquatic organisms, emphasizing the water chemistry. It is also one of the most progressively accepted models for assessing toxicity in aquatic life under the category of a quantitative tool with practical utility in water quality risk assessments [50,54,55]. This model is also helpful in toxicity assessment with biotic ligands in the context of the competitive binding of potential and protective cations inside the organism body. This model framework can lead to specific concrete results and explanations for the observed effects relating to the natural organic matter, aquatic life, and metal toxicity. Some studies have claimed to use a BLM to protect freshwater invertebrates based on data from freshwater fishes. Despite being one of the most reliable tools in water toxicology, a BLM also has its limitations and uncertainties. One disadvantage of using a BLM is that it is believed to be lacking in data [56]. Earlier, a gill surface interaction model (GSIM) was also introduced in 1983 by Gordon K. Pagenkopf [46]. The GSIM is used to evaluate certain measures of the acute toxicity of metals centered on fish gills and present results based on chemical and biological observations.

The endpoints studied throughout the progression of writing this review paper were as follows: (a) the health effects of copper metal ions and CuNP concentrations (low to high) on fishes and the environment; (b) the physiochemical effects of copper, copper metal ions, and CuNPs on fishes; (c) the effect of free copper; (d) the effects of copper complexes with other ligands on their bioavailability; (e) the effect of CuNPs in general; (f) and the effects of copper metal ions and CuNPs on different fish organs. A lot of research papers have suggested that in order to better understand the toxicity of copper and CuNPs, it is necessary to understand the mechanism by which copper impacts chemical and biological processes in the environment, specifically in fish. Therefore, the physiochemical parameters (temperature, pH, concentration, and dosage) of water bodies (lakes, ponds, rivers, etc.) in different geographic zones play crucial roles in this analysis.

**Figure 1:**
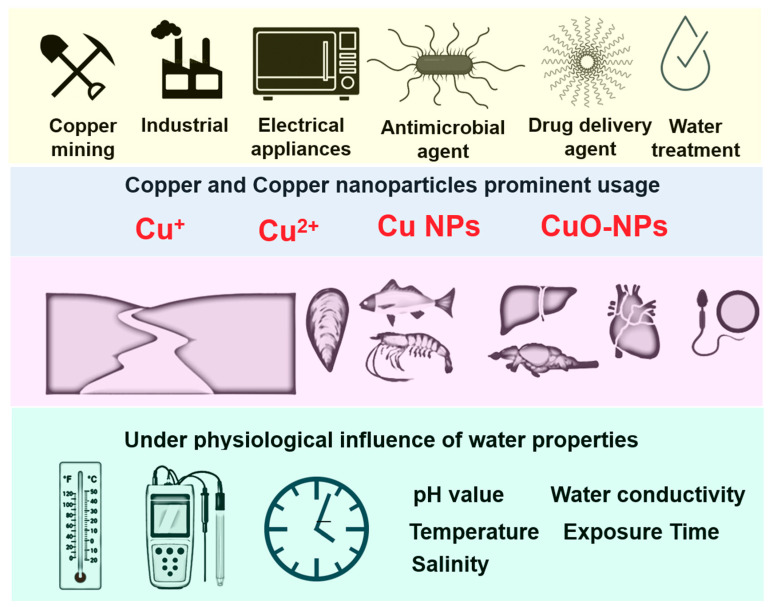
The schematic diagram depicts the bioavailability of current and potential applications of copper and copper nanoparticles, the main forms of copper that interact with biota, and the major parameters that influence copper toxicity. The top yellow panel shows the potential sources of copper and copper nanoparticle (CuNP) pollution that are generated in our daily lives. The middle blue panel shows the potential form of copper pollution in terms of Cu(I), Cu(II), Cu nanoparticles, or CuO nanoparticles. The middle pink panel shows the copper and copper nanoparticles that are released into water and subsequently ingested by aquatic animals and deposited in internal organs such as the liver, brain, heart, and reproductive organs. The bottom green panel shows the physical properties of water, like the pH value, temperature, salinity, water hardness, and exposure time, which can influence the copper and copper nanoparticle toxicity.

For these reasons, this review aims to describe copper and copper nanoparticle (CuNP) toxicity based on a set of physical and behavioral parameters. Models of different fish species are addressed here in this review. It has been reported earlier that excessive copper inside the body does not ensure a greater accumulation of copper in body organs, as the copper may bind with cations and consequently affect the normal function of the cellular metabolism. An example where this mechanism can be seen is through studies that have demonstrated an interference of Na ion regulation, which has been associated with acute metal toxicity, where copper assimilation on fish gills has been shown to impede Na ion efflux and affect the Na^+^/K^+^-ATPase activity [57,58]. The schematic representation of copper and CuNPs bioavailability, its potential applications, interaction with biota and major parameters influencing copper toxicity are presented in Figure 1.

## 2. Overview of Copper-Induced Toxicity in Aquatic Model Organisms

Copper is typically found in natural aquatic environments at a low concentration. The analysis of copper toxicity in aquatic organisms is important in aquatic habitats, which are susceptible to copper pollution near the ultimate receptors of industrial and urban wastewater and atmospheric deposition via copper mining and smelting ores, thereby elevating the copper concentration in the aquatic ecosystem [44,59]. Copper at a level above the normal level required for growth and development in species can result in accumulation and cause irreversible harm [44,60,61,62,63,64]. Being non-degradable, copper is a potential toxicant that might build up in an environment, and its accumulation and release are of global concern [65,66]. Therefore, we picked the topic of the toxicity of copper and CuNPs in fishes to summarize the recently published data.

Exposure to low (180 µg/L of Cu^2+^), medium, and high levels (3200, 1000, and 560 µg/L) of Cu^2+^ has been shown to induce changes in the morphology of winter flounder (*Pseudopleuronectes americanus*) fish. Histological techniques and electron microscope analysis revealed fatty metamorphosis in the liver, necrosis in the kidney, destruction of the hematopoietic tissue, and changes in gills in the groups exposed to high and medium copper levels. The model organisms exposed to low levels of copper featured vacuolated epithelial layers, as observed using light microscopy and electron microscopy [67]. Similarly, sheepshead fish (*Archosargus probatocephalus*), when exposed to a toxic concentration of cupric ions in sea water (8.5 mg/L), showed signs of lethargy, incoordination, and even death. However, when the organs from these fishes were examined, e.g., the serum, gills, liver, and kidney, different intoxication stages for each organ were detected in each individual examination. The fishes also featured swollen and congested kidneys, blunt and thickened capillaries, and congested gill lamellae. Lastly, potassium in the serum was observed to have reached a level considered lethal for mammals [68]. Acute short-term and long-term bioassays of copper toxicity for brown bullhead fish (*Ictalurus nebulosus*) have demonstrated liver distress and morphological changes of the skin, liver, and gills during histomorphological and histochemical analysis [69]. The hematological and physiological changes in the blood of juvenile *Prochilodus scrofa*, when exposed to acute copper concentrations of 20, 25, and 29 µg/L in water with predetermined measures (pH 7.5, hardness 24.5 mg/L as CaCO_3_) for 96 h, included an elevation in hematocrit and red blood cells at 25 and 29 µg/L of copper concentration. In addition, an increase in leukocytes and potassium levels was also seen at a 29 µg/L copper concentration. Moreover, plasma sodium and chloride levels decline at a lower blood pH [70]. Tilapia fish (*Oreochromis niloticus*) (both sexes) reared in freshwater were exposed to 0.5, 1.0, and 2.5 mg/L of waterborne copper for a period of 21 days. After the exposure period of 21 days, the fish experienced lifting of the lamellar epithelia and intense vasodilation of the lamellar vascular axis in gills at a high concentration. There was also vacuolation and necrosis of the liver in the high concentration group, as revealed by histopathological tests [71]. After 42 days of dietary copper exposure (2000 mg copper/kg dry wt. feed) of *Oreochromis niloticus,* the amount of copper was elevated by up to 30-fold in the intestine, 3-fold in the liver, and 2.7-fold in the gills. The fishes indicated a reduction in food uptake and weight gain by 21 days of exposure, where the livers of the fishes were marked by hepatic lipidosis, suggesting redistribution of copper to the liver and delayed hepatotoxicity [72]. A similar pattern was found when juvenile rainbow trout (*Oncorhynchus mykiss*) were exposed to copper at low (11 µg/g), medium (300 µg/g), and high (1000 µg/g) concentrations incorporated in their diet for 28 days, where copper accumulated in the liver and gut tissue. A high elevation of copper content was also seen in the bile. This study discussed the evidence of hemostatic interaction between two routes of copper uptake and the uptake of waterborne copper across gills when pre-exposed to dietary copper [73]. In another study, the effects of endogenous cortisol levels (200 ng/mL) were compared to sublethal copper levels (1.9 µM). The exposure of freshwater common carp (*Cyprinus carpio*) was carried out both alone and in combination. The individually cortisol-exposed fishes showed increases in cortisol levels, with increases in Na^+^/K^+^-ATPase activity, plasma Na^+^, and plasma osmolarity, whereas the fishes with copper showed an anaerobic metabolism, gill damage, decreased Na^+^/K^+^-ATPase activity, decreased plasma ion levels, and blood thickening. Overall, the study suggested that the pretreatment of copper-exposed fish with cortisol partially protected these fish by reducing the copper-induced reduction in Na^+^/K^+^-ATPase activity, which for the first time proved the intermediate role of cortisol in the toxic effects of copper [74].

In another study, the toxicity of copper to crucian carp (*Carassius carassius*) in soft water was tested after exposing the fishes to a copper-rich medium with certain specified parameters (pH 6.6, conductivity 25 µs/cm, 2.91 mg Ca^2+^/L, approx. 300 µg Cu^2+^/L). The 300 µg Cu^2+^/L copper was not acutely toxic (96-LC50) to the fish, as mortality was observed after 10 days of copper exposure. The concentration of copper used in this study was ten times as high as the copper concentrations (10–20 μg/L) shown to be acutely toxic to other freshwater teleosts in soft water [75,76]. As early as the second day of exposure, an increase in hematocrit, plasma chloride, and sodium were observed among fish in the copper-rich medium. *C. carassius* has the unique ability to survive under prolonged anoxia; copper-exposed fish developed minor hypoxia, suggesting that the mechanism behind high copper tolerance is its ability to avoid hypoxia. On the other hand, the osmoregulatory disturbances indicated the ability of crucian carp to survive low plasma osmolality for a relatively long time. These observations showed that *C. carassius* has a higher tolerance to copper as compared to other fish species [77]. Another study analyzed the acute toxicity of copper in Senegalese sole (*Solea senegalensis*) using a static bioassay test. It was found out that after 96 h, the LC50 value of metal was 0.32 mg/L (cupric form). The sublethal concentrations of 0.01–0.1 mg/L of copper cause histological alterations in liver and gills, suggesting that the intensity of the increased histological alterations gradually increase with copper concentration and exposure time. The study concluded the persistence of sublethal effects and histology, therefore becoming a tool that can reveal sublethal effects of heavy metals on aquatic biota and environments [78]. This is somewhat related to another study, where the exposure of copper at 250 µg/L for 24 h in butterfish (*Poronotus triacanthus*) was compared to a subacute toxicity test with a copper concentration of 25 µg/L for 7 days. This study revealed increases in copper in the liver, kidney, gills, and muscle tissues, with major accumulation in tissues analyzed by atomic absorption spectroscopy. After the treatment period, the fishes were returned to normal water for 48 h for recovery, suggesting the severe effects of copper for the 7-day exposure group compared to the group exposed for 24 h. This indicates that copper toxicity is also dependent on the length of exposure [79]. A 96-h copper exposure study was conducted with *Prochilodus scrofa*. Gill damage was characterized by epithelial lifting, cell swelling, and chloride and mucous cell proliferation. Complete gill recovery occurred on the 45th day after transfer to clean water [80]. Based on the toxicity experiments conducted with diverse fish species, waterborne copper exposure can induce various kinds of organ damage in the gills, liver, kidney, brain, gonad, and heart. We carry out a more detailed discussion in the following section. An important fish species, *Rhamdia quelen*, was evaluated at different waterborne copper concentrations (2, 7, and 11 µg Cu/L) for 96 h. Leukocyte infiltration, hepatocyte vacuolization, and areas of necrosis causing raised levels of lesions were observed upon 7 and 11 µg Cu/L exposures, which were recorded during liver histopathological findings. In addition, damage to secondary lamellae on the gills started showing at the 2 µg Cu/L concentration, with the loss of microridges of pavement and hypertrophy at 7 and 11 µg Cu/L. Aside from these observations, an increased carbonic anhydrase activity was also compared from the 2 and 7 µg Cu/L groups. A significant disturbance in the osmoregulatory functions also implied that copper induces morphological, physiological, and biochemical effects for varying copper concentrations [81].

## 3. Overview of Copper-Induced Organ Toxicity in Elected Fish

**Gills**: The gills play an important role in detecting the effects of metal or any other substance, as they are in direct contact with the surrounding water. The large surface area of gills in the species *Prochilodus scrofa* has been shown to favor copper uptake from water [82]. *Oreochromis niloticus* fish were exposed to waterborne copper at levels of 40 and 400 µg/L and the gill Na^+^/K^+^-ATPase activity, plasma Na^+^, Cl^−^, osmolality, protein, glucose, and cortisol levels were detected to be dysregulated [83,84]. In other studies, copper affects fishes under different ranges of alkalinity and water hardness. These effects were demonstrated by the alteration of gill function, biochemical parameters, and osmoregulation capacity [68,83,85,86,87,88,89]. Similarly, gill histopathology showed a dose–response relationship with water copper levels relative to sex and mass [90], ages of the fishes [91], concentration and exposure time [78], and species variation [92].

**Liver**: In European seabass (*Dicentrarchus labrax*), copper was injected intraperitoneally and the metallothionein level in the liver was determined in the liver. Copper exposure was found to reduce the metallothionein level in the liver [93]. In another interesting study, common roach fish (*Rutilus rutilus*) were exposed to 80 µg/L of copper (sublethal concentration) for 7 days. These fish had fed and starved stages. The liver tissue of the starved fish showed significant accumulation and no significant change in copper content was noticed in the fed fish. Therefore, this suggests that the nutritional status of fishes plays a role in the toxicity responsiveness [94].

Another similar study was conducted. An incubation period of 14 days with a sublethal concentration of copper in zebrafish (*Brachydanio rerio*) was established, then the fish were left in clean water for another 14 days. However, after being in the clean water for 14 days, the livers of the fish still contained high levels of copper, with no reverse actions that took place. This suggests the need for more studies on the late stages of toxicity [95,96]. In another study, the effects of copper on the liver enzymes hexokinase, phosphofructokinase, pyruvate kinase, lactate dehydrogenase, and glycose-6-phosphate dehydrogenase in *Prochilodus lineatus* demonstrated the different effects of copper in relationship with temperature for all the mentioned enzymes [97]. Cytolysis, cytoplasm blebbing, focal necrosis, hemorrhaging within sinusoids, dilation fibrosis, cytoplasmic vacuolization, and pyknosis were observed when major South Asian carp (*Catla catla*) were exposed to sublethal concentrations of copper ions for three weeks [98]. The three-spined stickleback (*Gasterosteus aculeatus*) is shown in this study to be a suitable model to study the oxidative stress caused by the metals. When these fish were exposed to copper sulphate for three weeks, copper was seen to induce oxidative stress in the liver, even before the detection of copper accumulation in the liver. This suggests the role of copper in differential mechanisms during copper uptake and metabolism [99].

**Kidneys**: In fish, the kidneys play an important role related to hematopoiesis. When *Dicentrarchus labrax* was injected with copper, this activated the redox process and generated oxy-radicals but did not affect the catalase activity in vitro, while also increasing the malondialdehyde (MDA) levels (a marker for lipid oxidation) [100]. In common carp, copper has been identified to have a high binding affinity to the head kidney [101] and waterborne copper exposure can significantly reduce hematopoietic potential in the head kidney [102].

**Reproductive organs (gonads)**: The effect of heavy metal accumulation in male and female gonadal organs can have severe toxic effects on future generations. A study demonstrated that copper accumulation in gonad tissue of *Carassius carassius auratus* and *Xiphophorus helleri* increased linearly with the increase in sublethal levels of copper over a given period of exposure. Simultaneously, there was a reduction in the growth rate and reproductive performance in tested fishes [103]. In the same study, decreases in the mean diameter of eggs of *Carassius auratus* and the mean weight and body length of *Xiphophorus helleri* were reported. In another study, 30-day copper exposure gave evidence of copper accumulation in various organs in relation to age and dose dependency in silver sea bream (*Sparus sarba*) [104]. A high level of copper deposition in the liver and gonads was present in common carp (*Cyprinus carpio*) and *Rutilus ylikiensis* [105]. The copper accumulation in the rosy barb fish (*Pethia conchonius*) in a longer study duration of 2, 3, and 4 months in hard water resulted in the transient arrest of spermatogenesis after 2 months of exposure and the reappearance of spermatogenesis 3 months onwards. A maximum induction of atresia was also seen [106]. A high copper concentration in male testes and female ovaries from Torch Lake, Michigan, was observed, but no significant teratogenic effect was seen for the obtained larvae after reproduction [107]. The extent of copper accumulation was also attributed to the differences in the feeding and behavior of tilapia (*Oreochromis mossambicus*) and African sharptooth catfish (*Clarias gariepinus*) [108]. In a study to assess the pattern of accumulation of copper in aquatic organisms, two families of the fish species *Cyprinidae* and *Percidae* were analyzed during different stages with varying sexes, ages, and living environments. The quantity of copper rapidly increased in the gonads during the pre-spawning time of the fishes [109]. In the three-spined stickleback (*G. aculeatus*), scientists discovered that copper exposure at the parental generation can increase both T4 and T3 levels in eggs, suggesting copper exposure can potentially induce transgenerational endocrine disruption [110].

**Heart**: Biochemical and hematological parameter testing was conducted on *Oncorhynchus mykiss*, where copper sulphate (0.2 mg/L) was shown to induce a slight damaging effect to various tissues as indicated by measurements biochemical and hematological parameters such as glucose, aspartate aminotransferase (ASAT), alanine aminotransferase (ALAT), acetylcholine esterase (AChE), lactate dehydrogenase (LDH), hematocrit, and total protein. This toxicity was elevated with a high presence of sulfuric acid at pH 6.5 [111]. Copper sulphate was then studied for the serum, brain, heart, and muscle tissue in vivo for carp (*Cyprinus carpio* L.). The analysis demonstrated the inhibition of acetylcholine esterase (AChE) activity, which is considered an indicator of hazards in the natural environment [112]. For *Oncorhynchus mykiss*, the sensitivity to acute and chronic exposure to copper was ranked as follows: larval growth > heart rate > larval survival > embryo survival. A significant growth reduction of fish occurred at 0.015 mg Cu/L [113]. This observation clearly suggests a reduction in fish offspring quantity and quality, as fish embryos are sensitive to water pollution from early development stages. A 24-h exposure of *O. mykiss* to 4.9 µmol Cu/L in fresh water at pH 7.9 caused a rapid decline of plasma Na^+^ and Cl^−^ and arterial O_2_ tension, leading to identifiable tachycardia due to copper toxicity [114]. In a study where Danio rerio embryos were exposed to copper at a concentration of 11–1000 µg/L, a high concentration of copper led to faster heart rates at 28 h postfertilization, suggesting a stress response in fish embryos [115].

**Brain and behavioral changes**: A sublethal concentration 0.3 mg/L of copper ions was provided for the fish species *Catla catla* for 3 weeks, and clear spaces around nuclei, spongiosis, and the migration of mononuclear cells were observed on the third week [98]. The common carp *C. carpio* was challenged with 0.22, 0.34, and 0.84 µM of copper for a week, and both a significant decrease of brain serotonin and dopamine neurotransmitter contents associated with feeding behavior and locomotor control alteration were reported [116]. A sublethal concentration of copper (100 µg/L) demonstrated hyperactivity in *Archosargus probatocephalus* and *Arius felis* but had little effect on *Micropogon undulates* and no effect on *Lagodon rhomboids* [117]. In a similar study, an analysis focused on changes in the locomotory and exploratory behavior of the catfish *Arius felis*, both before and after copper exposure, where a low concentration (5 to 50 µg/L) of Cu^2+^ elicited hypoactivity and a high concentration (100–200 µg/L) caused hyperactivity after exposure [118]. To observe the shoaling behavior upon exposure to copper, Atlantic silverside fish (*Menidia menidia*) were used as model in another study. This study showed a relative decrease of distance from that of others within the same shoal group [119]. In another investigation, a reduction in the food intake of *Salmo gairdneri* at a copper concentration of 100–300 µg/L was observed, with a gradual return to normality in comparison to normal control fishes over a study parameter of 40 days [120]. The locomotor activity and feeding behavior in brook trout (*Salvelinus fontinalis*) also suggested no long-term effects on the species [121]. A study on bluegill (*Lepomis macrochirus*) analyzing foraging behavior was conducted with two different setups to assess the reaction distance and functional response between the model organism *Lepomis macrochirus* and a prey animal. This study concluded that a copper concentration of 18–28 µg/L may reduce fish growth in wild water bodies and alter their food patterns [122]. In an interesting study, copper was found to affect the chemosensations of benthos, zooplankton, and fishes, leading them to a metal-induced impairment of chemosensation, which is used to defend against predation and finding food and mates [123]. The avoidance behavior of the *Carassius auratus* was observed depending on the dissolved copper and temperature levels. The researchers specifically stated that fishes are more likely to stay in moderate temperature environments, as seen by the frequency of visits and time spent by fishes in this temperature of water [124]. In a long-duration study of 22 weeks of exposure of copper for *Lepomis macrochirus*, a reduction in growth, spawning, and survival rate was found [125]. Finally, one of the most significant conclusions we obtained from another study is that not all entities of rainbow trout in a water population are affected equally by the presence of copper in water [126]. This study highlights that fish behavior testing is very sensitive but has great individual variation, and a large sample size is considered necessary to obtain more definite conclusions. In addition, unified behavior test conditions and model fish species have to be set up in order to reduce intra- and intervariation. To reach this goal, our research group invented several versatile setups that are suitable for conducting multiple behavior tests on zebrafish for three-dimensional locomotion [127], novel tank exploration, mirror biting, predator avoidance, social interaction, shoaling [128], circadian rhythm [129], and short-term memory assessments [130].

The analysis of these papers revealed that copper exposure works differently under the influence of different external factors. The summary of selected fish, toxicity response, copper concentration and exposure time has been compiled in Table 1. We discuss some particular factors that play a significant role in copper toxicity mechanisms in the next section.

## 4. Effect of External Factors on Copper Toxicity in Aquatic Organisms

The quality of water is an important parameter in regard to copper toxic effects to aquatic organisms. In one of the studies, juvenile *Prochilodus scrofa* were habituated at 20 and 30 °C, with the pH maintained at 4.5 and 8.0. Later, the fish were exposed to copper for 96 h and the LC50 value was measured. Disregarding the water temperature, pH, and change in hematological variables, the fish displayed respiratory or ion regulatory disturbances resulting in an increased energy consumption for the restoration of hemostasis. This energy consumption is supposed to be utilized for basic physiological functions such as weight gain and growth requirements instead of restoring normal hemostasis [131]. In a similar study, *P. scrofa* was established to be highly sensitive to copper. It was regarded as a potential indicator for environmental monitoring. When the fish were restricted to a water with a pH ranging from 4.5 to 8.0, a pH of 4.5 was seen to be stressful for them. This low pH level causes changes in plasma glucose concentration levels. On the other hand, copper toxicity was higher for fishes kept in water at a pH of 8.0 in comparison to a pH of 4.5 [132].

A static test was employed to examine the acute toxicity of copper to *Capoeta fusca* in three different water mediums (soft, hard, and very hard, at 40, 150, and 380 mg/L of CaCO_3_, respectively). The results showed a reduction in copper toxicity with increments in water hardness, denoting more copper toxicity in soft water than that in hard water [133]. Furthermore, in another study, the acute exposure of copper at 5 µg/L displayed an increase in the metallothionine concentration and superoxide dismutase (SOD) activity in *Prochilodus lineatus*, suggesting that this might be the reason for lipid peroxidation in the liver and DNA damage in erythrocytes, along with the inhibition of muscle acetylcholine esterase (AChE) and some behavioral changes [134]. Similarly, metallothionine was established to be an effective biomarker in response to copper at different pH and dissolved O_2_ levels. These factors work together more effectively to produce significant disturbances in biomarkers rather than individually [135]. In the next study, juvenile *Salmo gairdneri* fish were exposed to 25–400 µg Cu/L for 24 h in a standardized environments of varying water hardness, with alkalinity maintained at a constant Na concentration. The results in high alkalinity water indicated a significant reduction in the effect of copper but no significant effect of increasing water hardness of copper on Na^+^ uptake or Na^+^ efflux, with no significant effect observed with the increase of water hardness. Alkalinity and water hardness had no effect on the apparent uptake of copper, but copper uptake was reduced by about 50% at a pH of 5.0 [85]. In another study, the effect of copper was identified in combination with elevated CO_2_ levels (hypercarbia) and also alone for small-scaled pacu (*Piaractus mesopotamicus*). These factors individually contribute to an increase in the liver factor lipid hydroperoxide concentration. However, this result was not replicated when these two factors were combined. Copper exposure alone elicited a hepatic superoxide dismutase activity, regardless of aqueous CO_2_ level, whereas the copper toxicity effect on glutathione peroxidase activity was dependent on the water CO_2_ levels. This study summarized branchial metallothionine and Na^+^/K^+^-ATPase as effective biomarkers for studying copper exposure. Additionally, the study emphasized that these biomarkers are not affected by water CO_2_ levels [136].

Copper has been a highly exploited metal for a very long time; hence, during the course of evolution, metal usage patterns have changed and toxicity results might vary under different sets of circumstances, which is why it is necessary to investigate all the dimensions of copper present in the current industry, including examining copper uses in various sectors, such as that for electrical and thermal conductivity uses, sensory devices, and biomedical and bioscience applications. This will help create a large amount of data that can be exploited to find useful qualities of copper for specific beneficial applications. We discuss the toxicity of CuNPs in the next section.

## 5. Effects of Copper Nanoparticles (CuNPs) on Fish

The various specific properties of CuNPs, such as their size, shape, higher surface to volume ratio, magnetism, high electrical and thermal conductivity, high melting point, oxidation reduction, catalytic activities, and low cost makes them preferred materials for a wide range of applications [137]. The appearance of CuNPs is usually brown or black as a powder [138]. The oxidation of copper tends to occur easily when exposed to air, resulting in the agglomeration of particles. To overcome this, CuNPs are either synthesized in an inert gas atmosphere [139] or coated with protective polymers and surfactants [140,141,142], also including organic and inorganic coatings, e.g., silica and carbon [143,144,145]. CuNPs are synthesized using “bottom-up” (chemical methods) and “top-down” (physical method) techniques. CuNPs applications are still being explored for use in biomedicine, bioscience [138], heat transfer systems [146], catalysts [147], antimicrobial materials [148,149], and sensors [150]. CuNPs demonstrate astonishing results as antibacterial and antimicrobial agents [151]. The metal ions release in a solution and in close proximity to microbial membranes. CuNPs tend to release Cu^2+^ from them, which in turn can create hydroxyl free radicals, damaging any membrane they interact with [152,153]. Therefore, to benefit from the reassuring qualities of CuNPs, the toxicity mechanism needs to be assessed in various different conditions and parameters to identify safe limits of usage for these CuNPs.

Considering the rampant usage of nanoparticles and the susceptibility of aquatic habitats to Cu pollution (because of being the ultimate receptor of urban and industrial waste, storm water run-off, and atmospheric deposition [83]), the analysis of toxicity patterns of CuNPs is important. The acute toxicity of soluble copper and 80-nm CuNP suspensions have been analyzed and compared in the zebrafish *D. rerio* in a recent publication [154]. The acute toxicity of CuNPs to *D. rerio* came out to be 1.5 mg/L 48 h LC50 and histological and biochemical data revealed that the primary organ for CuNP toxicity was the gills. CuNPs exposed to *D. rerio* at high concentration of 100 µg/L demonstrated different morphological effects and global gene expression patterns [154,155]. Similarly, in recent studies, the investigation of exogenous copper on intestinal development in zebrafish embryos was investigated. The application of 0.10 mg/L of CuNPs or Cu^2+^ damaged the zebrafish intestinal development, including thinning the epithelial cells, as well as shortening and reducing the number of intestinal villi. CuNPs and the release of Cu^2+^ on the intestinal development of zebrafish mutants *cox17*^−/−^ and *atp7a*^−/−^ were used to analyze the effects of a deficiency of copper on the dermis and intestinal innate immune system. The defective occurrence of intestinal development defects in copper-stressed embryos was detected through transmission electron microscopy (TEM) and hematoxylin and eosin (H&E) staining, where intestinal developmental defects via induction of endoplasmic reticulum (ER) and reactive oxygen species (ROS) stresses were observed [155]. Additionally, in another study, zebrafish embryos CuNPs (25 nm, 1 mg/L), soluble Cu, and polystyrene (PS) Nps (25 nm, 10 mg/L) exhibited innate immune responses focused on skin cells and intestines as likely organs of interaction. The mRNA expression of the immune responsive genes interleukin 1 beta (*il*1B) and immunoresponsive gene 1-like (*irg*1L) of CuNP-exposed embryos were observed to be weaker in intestinal tissue compared to rest of body, including the strong outer epithelium response. The nanoparticles were observed to accumulate in cavities of lateral neuromasts in the skin, increasing the expression of *il*1B locally [156].

Subsequently, chemical species of copper were established as governing factors for the acute toxicity of CuO-NPs [157]. For *Oncorhynchus mykiss*, when exposed to copper sulphate and CuNPs in a semistatic aqueous exposure regime, a mortality of 85% was seen on the 4th day at 100 µg/L of copper sulphate. This is greater in comparison to the 14% mortality resulting from CuNPs with the same parameters. The gills were observed as the main accumulation point of copper. In addition, CuNPs also induced ionoregulatory toxicity (decrease Na^+^/K^+^-ATPase activity), making them less acutely toxic than with an equal concentration of copper sulphate [158]. The copper content increased in all tissues in both forms of CuNPs and copper sulphate once exposed to juvenile orange-spotted grouper (*Epinephelus coioides*). Both forms also caused tissue oxidative stress and cell apoptosis. A comprehensive analysis revealed that dissolved copper is more toxic than CuNPs [159].

## 6. Overview of Organ Toxicity in Fish Induced by Copper and Copper Oxide Nanoparticles

**Gills**: The gills play a significant role in the bodies of fish, providing gaseous exchange and accumulating important nutrients from water bodies, CuNPs (40 µg/L) increased H^+^ and Na^+^/K^+^ pump activity in freshwater teleosts (*Prochilodus lineatus)* and made the exposed fish anemic; however, this effect was less pronounced in comparison to the copper salts [160]. CuNPs at a 10 µg/L exposure to euryhaline killfish (*Fundus heteroclitus)* in fresh and brackish water increased oxygen consumption and the aerobic scope in brackish water killfish but reduced Na^+^/K^+^-ATPase activity by >40% [161]. Exposure to copper sulfate at 1.5 mg/L and then CuO-NPs at 200 mg/L showed higher influences on growth indices, survival, and pathological signs of the gills of grass carp fingerlings after 30 days of exposure [162]. In another study, CuNPs at concentrations of 0.1, 0.2, and 0.5 mg/L in Caspian roach (*Rutillus caspicus*) demonstrated hyperplasia, fusion, and the detachment of secondary lamellae; reduction in length of secondary lamellae; and cellular degeneration in gills [163]. Similarly to detachment, sticking of attached lamella, and hyperplasia were detected at 80 mg/L, while aurism, inflation of squamous cells, and shortening of secondary blades were detected at 10 and 40 mg/L after exposure of 6 weeks of common carp (*C. carpio*) to CuONPs [164]. *C. carpio* specimens weighing 40–45 g were exposed to three sublethal doses of waterborne engineered Cu-NPs (0, 0.5, 1, or 1.5 mg/L) for a period of 14 days. The gill tissue showed degenerative secondary lamella, fused lamella, necrosis, and edema in common carp *(C. carpio)*, induced via the alteration in gill histology and oxidative stress parameters in a dose-dependent manner [165]. CuNPs were less toxic than dissolved copper for the two studied fish species, namely dwarf cichlid *(Apistogramma agassizii)* and cardinal tetra *(Paracheirodon axelrodi).* Fishes were exposed to 50% of the LC50 for CuO-NPs (dwarf cichlid, 58.31 μg/L; cardinal tetra, 69.6 μg/L) and Cu (dwarf cichlid, 20 μg/L; cardinal tetra, 22.9 μg/L) for 24, 48, 72, and 96 h. Oxidative stress was promoted in the dwarf cichlid [166]. CuNPs were also shown to be toxic to tilapia, a freshwater edible fish, in comparison to dibutyltin at an exposure concentration of 15 mg/L, inducing oxidative stress and hindering fish growth and development [167].

**Liver**: CuNP exposure at concentrations of 0.1, 0.2, and 0.5 mg/L in Caspian roach *(R. caspicus)* induced prominent changes in the liver upon histological analysis, showing blood congestion in central veins, cellular hypertrophy, necrosis of the hepatocytes, and nuclear pyknosis [168]. Similarly, common carp *(C. carpio)* exposed to 20 and 100 µg/L of CuNPs in accordance with the earlier stated study exhibited liver damage, which manifested as cells showing pyknotic nuclei. In addition, proteomics analysis revealed downregulation of several proteins (e.g., ferritin heavy chain and cytoglobin-1) and the upregulation of diphosphomevalonate decarboxylase and selenide, indicating deleterious effects in the tissues studied, which may affect fish growth and development [169]. Exposure of *T. fasciatus* to CuNPs at 20 and 100 µg/L caused a dose-dependent increase of copper in the liver, with an increase in oxidative stress indicators, malondialdehyde (MDA), total superoxide dismutase (T-SOD), glutathione (GSH), catalase (CAT), and activities of caspases in the liver [170]. To analyze the toxicity of copper nanoparticles (CuNPs) over traditionally dissolved copper, *O. mykiss* fish were exposed to CuNPs. This led to hepatitis-like injuries and cells with pyknotic nuclei in the liver [96].

**Kidney**: Exposure of Caspian roach *(R. caspicus)* to CuNPs at concentrations of 0.1, 0.2, and 0.5 mg/L induced histological changes in the kidney, including severe degeneration of the tubule cells, interstitial tissue, glomerular shrinkage, and an increase in interstitial tissue cells and macrophage aggregation [163]. At 10, 50, and 100 µg Cu/L copper concentrations, copper accumulated in the kidney with both CuSO_4_ and CuO-NPs, while CuO-NPs were more effective than CuSO_4_ in tissue accumulation and affecting liver enzyme activity [171]. When common carp *(C. carpio)* was exposed to CuNPs at 0.25 mg/L and to CuSO_4_ at 25 mg/L, both copper forms damaged the liver and kidney, while CuSO_4_ caused more severe damage in common carp in comparison to CuNPs [172].

**Reproductive organs**: CuO-NP exposure in mature guppies *(Poecilia reticulate)* and larvae was analyzed in two different tests of acute and chronic toxicity at concentration ranges of 0.5–45 mg/L for 96 h and 0.5–10 mg/L for 8 weeks, respectively. The 10 mg/L chronic exposure affected reproduction, lowered reproductive stress, prolonged parturition time, and increased the mortality rate, indicating toxicity for both mature fish and larvae [173]. CuO-NPs had negative impacts on the freshwater organisms and *Hyphessobrycon eques*, specifically on feeding, reproduction, and survival, with an increase in ROS, representing the potential CuO-NP effect on fresh water species [174]. In another study, CuNPs and CuSO_4_ were examined with adult male catfish *(Clarias batrachus)*, where testis-related genes showed regulation, an increase in the level of androgens, and disruption in the structural analysis [175].

**Heart**: In a study on early developmental stages of zebrafish, the exposure to CuO-NPs resulted in the prevention of looping of the heart tube during cardiogenesis, also disturbing dorsoventral patterning and increasing *wnt8* and *vent* expression, indicating that CuO-NPs might exert developmental toxicity [176].

**Brain and behavioral changes**: The freshwater edible fish tilapia, when exposed to CuNPs and dibutyltin for a short period, demonstrated that the oxidative stress enzymes glutathione (GSH), glutathione-s-transferase (GST), and acetylcholine esterase (AChE) were reduced in the brains of treated fish groups, where CuNPs and dibutyltin caused oxidative stress and imparted serious deleterious effects on tissues. This may affect the development and growth of fish, hence confirming CuNPs as being more toxic in comparison to dibutyltin [167]. In another experiment, when juvenile rainbow trout *(O. mykiss)* were exposed to CuSO_4_ and CuNPs (20 or 100 µg/L), histological analysis revealed broad organ injuries, which were similar in both CuSO_4_ and CuNPs. Some mild changes were observed in the brain. The researchers concluded that CuSO_4_ and CuNPs caused a similar kind of toxicity, but the severity of injuries caused to the brain with CuNPs was greater than that with an equivalent concentration of CuSO_4_ by the end of experiment [92]. In another study, free swimming zebrafish larvae were exposed to CuNPs and exhibited reduced lateral line neuromasts (LLN) and a reduced performance of rheotaxis, which is important for the survival and development of zebrafish [177]. The juvenile rainbow trout, when exposed for 12 h to 50 µg/L of CuNPs or CuSO_4_, resulted in different effects. CuNPs caused a significant increment in the ratio of oxidized to reduced glutathione in the brains of fish, indicating oxidative stress, which was not seen with CuSO_4_. This study specified that CuNP toxicity might be due to a mechanism distinct from the metal salt [178]. The summary of selected fish, toxicity response, tested form of CuNPs characteristics, concentration and exposure time has been compiled in Table 2.

## 7. Effect of External Factors on CuNP and CuO-NP Toxicity in Fish

Physiochemical factors influence the toxicity of CuNPs in aquatic organisms, such as the surrounding water medium, dissolved organic substances, pH, temperature, and salinity [182,183]. In another study, *O. mykiss* fish were exposed to CuO-NPs at concentrations of 1, 5, 20, and 100 ppm. The physiochemical parameters of the water were kept constant, such as a temperature of 22 ± 2 °C, an oxygen saturation of 90.9 ± 0.2%, a pH of 7 ± 0.004, maintaining the same concentration of CaCO_3_. After 96 h of exposure, no mortality was observed amongst the fish; however, the counts of white blood cells, eosinophils, neutrophils, hematocrits, and lymphocytes were affected, without any significant effects on monocytes and hemoglobin. The study concluded the inclusive hardness (270 ppm) nullified the lethal effect of copper on *O. mykiss* [184]. The toxicity of nanosized-Cu was studied under the criteria of size distribution and solubility in an E3 medium, demonstrating retarded hatching, morphological malformation, and even mortality after 96-h postfertilization in zebrafish embryos. Additionally, 0.1 mg/L of nanosized Cu was found to have more toxicity than 0.06 mg/L of Cu^2+^. The study indicated that nanosized Cu aggregates and forms of Cu released from nanosized Cu might play a combined role in causing toxicity to zebrafish embryos; however, in lieu of data, this fact cannot be established as of now [179].

CuNP temperature-dependent toxicity was observed for *O. mykiss*, *P. promelas*, and *D. rerio.* At high temperatures (26 ± 1 °C), CuNPs showed enhanced aggregation and a high rate of dissolution in comparison to low temperatures (15 ± 1 °C). The paper also suggested that the intrinsic physiology between fish species may also play a role in explaining the difference in sensitivity to CuNPs [183]. In another study, the effects of soluble copper and CuNPs were examined in zebrafish. The result demonstrated acute toxicity of CuNPs to zebrafish at 48 h LC50 of 1.56 mg nanocopper/L. Although gills were revealed to be the primary organ which was affected by aggregation of CuNPs in water, the research group specified that toxicity cannot be verified by the dissolution of particles alone [154]. In the next study, *O. mykiss* fish were exposed to CuNPs and CuSO_4_ at concentrations of 20 and 100 µg/L, where 100 µg/L of Cu as CuSO_4_ showed 85% mortality in comparison to 14% mortality for the CuNP group. Overall, CuNPs showed similar toxicity effects to CuSO_4_, occurring at lower tissue Cu concentrations than expected for the dissolved metal [158]. In a similar study, when the effects of CuO-NPs (in house-synthesized and commercial) and ionic copper (CuSO_4_) were studied on *L. pictus* embryos, CuO-NPs internalization and differential dissolution lead to developmental abnormalities. The synthesized CuO-NPs showed higher toxicity and increased dissolution (effective concentration (EC50) = 450 mg/L of copper, 2.5% by weight over 96 h, respectively) in comparison to commercial nanosized CuO (EC50 = 5395 mg/L of copper, 0.73% dissolution by weight over 96 h, respectively). It was suggested that the physiochemical properties of different forms of copper play essential roles in the toxicity mechanism of *L. pictus* embryos [180]. Further, in another study, six Cu particles, nanosized Cu and CuO, micron-sized Cu and CuO, and nano-Cu(OH)_2_-based fungicide (CuPRO and Kocide) were used in a septic tank system to analyze the fate, transport, and transformation of Cu particles in a decentralized wastewater treatment system, with a specific focus on the fungicides CuPRO and Kocide. The results demonstrated that Cu dissolution played a key role in determining the hazard potential of received particles, where the transformation of these materials in the septic tank rendered Cu as non-bioavailable to zebrafish embryos and prevented any effect on the hatching system. However, nanoscale materials showed 50% hatching when above 0.5 ppm and micron-scale particulates with no effect on hatching until reaching 10 ppm. The addition of carbon components such as humic acid in this study lead to a dose-dependent decrease in Cu toxicity, as determined using a high content zebrafish embryo screening assay [185]. Next, in a study on the effect of CuO-NPs, toxicity was detected for *Cyprinodon variegatus* at various salinity regimes. This involved two sets of experiments. In experiment 1, the fish were acclimated to hyposmotic, isosmotic or hyperosmotic salinity for 14 days and then exposed to copper at 16.6 µM Cu^2+^ for 12 h. In experiment 2, the fish were exposed to 14.6 µM Cu^2+^ for 6 h after 14 days of salinity acclimation. As a result, the fish acclimated to a 2.5 ppt salinity were more sensitive to Cu than those acclimated to 10.5 or 18.5 ppt of seawater; the same 2.5-ppt-acclimated fish were markedly affected by Cu, increasing the whole body Cu and liver lipid peroxidation (LPO) and decreasing whole body Na levels, respectively.

The CuO-NPs also caused behavioral changes in the fish, an increase in mucus secretion, and a loss in equilibrium. This study demonstrated that in euryhaline fish, salinity acclimation has a drastic effect on Cu toxicity [181]. The significant outcomes on the toxicity of copper and copper nanoparticles (CuNPs) for the organs of studied fish are summarized in Table 3.

## 8. Discussion and Conclusions

The ever-increasing metal usage in different forms around the world is a matter of great concern in present times, as it eventually affects all forms of life in our ecosystem. Therefore, it is important to understand the underlying chemistry and mechanism of these metals to the environment and organisms at a basic phenomenal level. In this review article, we have compiled the data related to copper metal, CuNP, and CuO-NP toxicity to various fish. Most of the waste products from different industries, mining sites, and other human activities enter water bodies through various sources, including via soil erosion, weathering, water transportation, and other human activities. Hence, flora, fauna, and aquatic organisms are considered to be the most effective indicators for studying water pollution and toxicity levels in order to analyze the rise of pollution in regard to changing environmental measures. The current research patterns will help to pave the way for future research trends. A large-scale data compilation of similar lines might help to check the safety criteria for usage of these copper particles and also set safe environment regulations. Considering that aquatic model organisms are cheap and easy to maintain, we have compiled data related to them. We believe that vertebrate aquatic model organisms such as zebrafish might be used as potential screening organisms to study the effects of copper metal ions, CuNPs, and CuO-NPs with different study parameters to lead us to specific results and collect a large amount of data to further examine other relevant model organisms.

It is very important to understand the effects of copper and CuNPs on affected organisms. However, the data related to CuNPs are limited and their toxicity is not completely understood. Some studies have demonstrated CuNPs to be less toxic than CuSO_4_ [172], other studies CuO-NPs have been demonstrated to be more toxic than CuSO_4_ [171], whereas others have reported that CuNPs have a similar toxic effect to CuSO_4_ [158], making it difficult to obtain established and concrete results. The physiochemical characteristics of surrounding water mediums, such as in aquaria, ponds, lakes, and seas, also affect the Cu dissolution, speciation, and toxicity, in addition to the dosage, physiological concentrations, and internal structure of aquatic organisms. Although research on copper toxicity has been prevalent for many years now, the investigation of CuNP toxicity started recently, driven by the high usage of metal nanoparticles in consumer products. It is important to understand the toxicity mechanisms in order to establish guidelines and ensure safe usage of these nanoparticles, such that aquatic organisms are not severely affected and remain safe for human consumption.

## Figures and Tables

**Table 1:** Summary of copper ion toxicity in fish based on target organs.

Organs	Model Organism	Toxicity Response	Copper Concentration	Exposure Time	References
Gills	*Oreochromis niloticus*	Inhibition of Na^+^/K^+^-ATPase activity	Cu^2+^, 40 and 400 µg/L	0, 3, 7, 14, and 21 days	[83]
	Oncorhynchus *mykiss*	Inhibition of Na^+^/K^+^-ATPase activity (LC50 10 µM)	1 mM	30 min	[88]
	*Oncorhynchus* *mykiss* *Perca flavescens*	Different mechanisms of copper tolerance or copper burden do not necessarily translate into toxicity. LC50 Hard Water 1.05 vs. 4.16 mM. LC50 Soft Water 0.10 vs. 0.44 mM	CuSO_4_. Hard water: 0.38 to 15.35 µM. Soft water: 0.11 to 0.77 µM	3 h	[92]
	*Oncorhynchus mykiss*	Decline of plasma Na^+^ and Cl^−^ and arterial O_2_ tension, cell swelling, thickening, and curling of the gill lamellae	4.9 µmol/L	24 h	[114]
	*Oncorhynchus mykiss*	Hyperplasia, aneurisms, and necrosis in secondary lamellae of the gills	CuSO_4_, 20 or 100 µg/L	0, 4, and 10 days	[96]
	*Opsanus beta*	No inhibition of Na^+^/K^+^-ATPase	Copper, 12.8 ± 1.6 µM, 55.2 ± 5.0 µM	30 days	[89]
Liver	*Rutilus rutilus*	Combined effect of nutrition and copper in toxicity	80 µg Cu/L 0, 25, 100, and 200 µg/L, and 20 or 100 µg/L	7 days	[94]
	*Brachydanio rerio*	Copper accumulation. Large lysed area, hepatocyte alterations, and increase in antioxidative defense	CuSO_4_, 40 ± 5 or 140 ± 30 µg Cu/L	14 days	[95]
	*Oncorhynchus* *mykiss*	Hepatitis injuries and cells with pyknotic nuclei in the liver	CuSO_4_, 20 or 100 µg/L	0, 4, and 10 days	[96]
	*Prochilodun lineatus*	Activity of key liver enzymes change with ambient water surroundings. Copper accumulation in the liver was high in fish at 20 °C and pH 8.0	98 ± 0.8, 16 ± 0.2, 88 ± 0.8, 14 ± 0.5 µg Cu/L (pH 7.0, 4.5, 8.0). Temperatures of 20 and 30 °C	96 h	[97]
	*Catla catla*	Cytolysis, necrosis, pyknosis, and fibrosis at 24 h. LC50 0.75 mg/L	0.1 and 0.3 mg/L	3 weeks	[98]
	*Gasterosteus aculeatus*	Copper accumulation at day 8 and 12, induced oxidative stress	0, 25, 100, and 200 µg/L	4, 8, 12, and 21 days	[99]
	*Dicentrarchus labrax*	Metallothionine reduction with copper injection, depicting toxicity	500 ng/g	24 h	[93]
Kidney	*Oncorhynchus* *mykiss*	Damage to epithelium of some renal tubules and increased Bowman’s space	CuSO_4_, 20 or 100 µg/L	0, 4, and 10 days	[96]
	*Dicentrarchus labrax*	Damage to pronephros and lysosomal membranes. Oxi-radical generation. Copper depicted a high binding affinity with the head kidney of common carp. LC50 2500 ng/g	Cu injection, 50, 250, and 1000 ng/g	48 h	[100]
	*Cyprinus carpio L*	Buffering capacity of copper was high in fish. Increase in number of blast cells, proliferating cell nuclear antigen (PCNA), and apoptotic cells. Similar effects for short term and long term exposure	Short term: 0.75 mg/dm^3^ Cu. Long term: 0.075 mg/dm^3^ Cu	Short term: 3 h. Long term: 4 weeks	[101,102]
Reproductive organs	*Carassius auratus* and *Xiphophorus helleri*	Copper accumulation, effect on growth rate, and reproductive performance. Reduction in mean diameter and weight of eggs. 96 h LC50 0.30 and 0.36 ppm	0, 0.15, 0.20, 0.25, 0.30, 0.35, and 0.40 mg/dm^3^. 0, 0.18, 0.24, 0.30, 0.36, 0.42, 0.48, and 0.54 mg.dm^3^	96 h	[103]
	*Sparus sarba*	Accumulation was dose-dependent with similar accumulations in fingerlings and subadults; 24 h, 48 h, 72 h, and 96 h LC50 of 2.01, 1.28, 1.17, 1.03 mg/L for fingerlings and 2.36, 1.52, 1.34, and 1.24 mg/L for subadults, respectively	0.15, 0.30, and 0.45 mg Cu/L	30 days	[104]
	*Cyprinus carpio, Silurus aristotelis, Rutilus ylikiensis,* and *Carassius gibelio*	*C. carpio* and *R. ylikiensis* presented the highest metal content in gonads	Fish from Lake Pamvotis	-	[105]
	*Puntius conchonius*	Copper interfered with spermatogenesis temporarily, induced atresia in ovaries; 96 h Median Tolerance Limit (TLm) 0.571 ± 0.020 mg/L	Lake Nainital	2, 3, 4 months	[106]
	*Cyprinus carpio, Carassius auratus gibelio, Rutilus rutilus heckeli, Abramis brama, Aristichthys nobilis, Hypophtalmichthys molitrix,* and *Sander lucioperca*	Accumulation pattern was similar in the investigated species and increased greatly in the prespawning period	Freshwater ecosystem of Moldova	-	[109]
	*Gasterosteus aculeatus*	Copper increased T4 and T3 level in ovaries	100 µg/L	2 h, 1 week	[110]
	*Danio rerio*	Hatching inhibition and slow development	50–1000 µg Cu/L, 68.35 ± 4.27 and 244.36 ± 17.40 g Cu/L	48, 72, and 96 hpf	[115]
	*Lepomis macrochirus*	Survival was reduced, growth was retarded, and spawning was inhibited; 96 h TL50 1100 µg Cu/L. Maximum Acceptable Toxicant Concentration (MATC)/96 h TL50 0.02 and 0.04	40–162 µg Cu/L	22 month	[125]
Heart	*Cyprinus carpio L*	Increase in acetylcholine esterase (AChE)	1.0, 10.0, or 50.0 ppm	2 h	[112]
	*Oncorhynchus* *mykiss*	Heart rate decreased, LC50 1.33 to 0.06 mg Cu/L. Measured Toxicity Equivalency Concentration (TEC) 0.09, 0.04, 0.02 mg/L, copper. No Observed Effect Concentration (NOEC) 0.06, 0.03, 0.015 mg/L, respectively	0.008 mg/L to 8.0 mg/L	96 h and 65 days	[113]
	*Oncorhynchus* *mykiss*	Doubling of mean arterial blood pressure and cardiac failure	4.9 µmol copper/L	24 h	[114]
	*D* *anio* *rerio*	Fastest heart rates at 28 hpf for stress response	50–1000 µg Cu/L, 68.35 ± 4.27 and 244.36 ± 17.40 g Cu/L	24, 48, 72, and 96 hpf	[115]
Brain	*Cyprinus carpio*	Decrease in brain 5-HT and dopamine levels, affecting behavior and locomotor control	0.22, 0.34, and 0.84 µM	1 week	[116]
	*Lagodon rhomboids,* *Micropogon undulates,* *Archosargus probatocephalus,* and *Arius felis*	Behavioral variables, general activity, swimming speeds, and angular orientation of movements affected. Locomotor activity and angular orientation of movements	0.1 mg/L	72 h	[117]
	*Arius felis*	Here, 0.005, 0.01, and 0.05 mg, Cu^2+^/L hypoactive, 0.1 and 0.2 mg Cu^2+^/L elicited hyperactivity and an increase in orientation angle of movement	0.0, 0.005, 0.01, 0.05, 0.1, or 0.2 mg Cu^2+^/L	72 h	[118]
	*Saluelinus jontinalis*	Increase locomotor activity and decrease in feeding response	6–60 µg/L	2 h	[121]
	*Menidia menidia*	At low copper concentrations, an increased swimming speed, decreased rate of change of direction and their nearest-neighbor distances. Swimming in a parallel orientation were observed; 96 h LC50 136 µg/L Cu	0–100 µg Cu/L	4 days	[119]
	*Salmo gairdneri*	Increased swimming speed	0–200 µg Cu/L	96 h–30 days	[120]
	*Lepomis macmchirus*	Decreased prey consumption and increased handling times of preying on five different invertebrate prey	31, 1 80, 1 710 µg/L	1 week	[122]
	*Pimephales promelas*	Copper may disrupt important developmental stages in the embryonic olfactory system, also chemosensory impairment	0 or 10 μg Cu/L	5–7 days	[123]
	*Carassius auratus*	Frequency of entry and time spent increased at a temperature of 21.5 ± 0.1 °C	0.010 ppm, temperatures of 21.1 ± 0.1 and 21.5 ± 0.1 °C	24 h	[124]

**Table 2:** Summary of copper nanoparticle toxicity in fish based on target organs.

Organs Affected	Organism	CuNPs Tested	Adverse Outcome	Time	Characteristic	References
Gills	*Danio rerio*	CuNPs	Toxicity in gills, not adequately explained by dissolution of particles alone; 48 h LC50 1.5 mg/L	48 h	80 nm	[154]
	*Oncorhynchus mykiss*	CuNPs	Reduction in branchial Na^+^/K^+^-ATPase, increase in Thiobarbituric acid reactive substances (TBARS)	0, 4, 10 days	87 ± 27 nm, 20 and 100 µg/L	[158]
	*Fundulus heteroclitus*	CuNPs	>40% reduction in Na^+^/K^+^-ATPase in FW	48 h	5–10 nm, 10 µg/L	[161]
	*Ctenopharyngodon idella*	CuO-NPs	Growth reduction	30 days	50 nm, 100 and 200 mg/L	[162]
	*Cyprinus carpio*	CuO-NPs	Cell swelling, aourtism, edema, hyperplasia	6 weeks	10, 40, 80 mg/L	[164]
	*Apistogramma agassizii*	CuO-NPs	Oxidative stress	96 h	HD 50 nm, 58.31, 69.6 µg/L	[166]
	*Plotosus lineatus*	CuNPs	Increase in H^+^ and Na^+^/K^+^ pump activity	96 h	40–60 nm, 12.16 ± 1.77 µg/L	[160]
	*Rutilus rutilus caspicus*	CuNPs	Fish became anemic	21 days	40 nm, 0.1, 0.2, and 0.5 mg/L	[163]
	*Ceriodaphnia silvestrii* and *Hyphessobrycon eques*	CuO-NPs	Fusion of secondary lamellae, cellular hyperplasia. Increase in reactive oxygen species (ROS). Apoptosis and necrosis	Acute-24 h Chronic-8 days	HD 30 nm, acute, 0, 0.7, 10.0, 13.0, 16.0, 19.0 µg/L, chronic, 0, 0.5, 1.0, 2.0, 4.0, 8.0, 10.0 µg/L	[174]
Liver	*Rutilus rutilus caspicus*	CuNPs	Deformation of nuclei, cytoplasmic vacuolation, cellular degeneration, congestion in the blood sinusoids, and necrosis of the hepatocytes	21 days	40 nm, 0.1, 0.2, and 0.5 mg/L	[163]
	*Rutilus rutilus caspicus*	CuNPs	Blood congestion in central veins and necrosis of hepatocytes. Pyknotic nuclei	21 days	0.1, 0.2, and 0.5 mg/L	[168]
	*Cyprinus carpio*	CuNPs	Increase in oxidative stress markers MDA, T-SOD, GSH, CAT, and caspases	7 days	<50 nm, 20, and 100 µg/L	[169]
	*Trachidermus fasciatus*	CuNPs	Increase in MDA, T-SOD, CAT, GSH, Casp-3, 9, decrease in Na^+^/K^+^-ATPase, and cyt c	30 days	10–30 nm, 20 and 100 µg/L	[170]
	*Oreochromis niloticus*	CuO-NPs	Increase in SOD, CAT, GPX	1, 7 and 15 days	<50 nm, 10, 50 and 100 µg/L	[171]
Kidney	*Rutilus rutilus caspicus*	CuNPs	Degeneration in the tubule cells, interstitial tissue, glomerular shrinkage, increase in interstitial tissue cells, and macrophages aggregation	21 days	40 nm, 0.1, 0.2, 0.5 mg/L	[163]
	*Oreochromis niloticus*	CuO-NPs	Excess copper bound to metallothionine (MT)	1, 7 and 15 days	<50 nm, 10, 50, and 100 µg Cu/L	[171]
	*Cyprinus carpio*	CuNPs	Tubular vacuolization and necrosis, melano–macrophage center, Bowman’s capsule edema, glomerulus degeneration, and hyperemia	14 days	40 nm, 0.25 and 25 mg/L	[172]
Reproductive Organs	*Poecilia reticulata*	CuO-NPs	Lowered reproductive stress, prolonged parturition time, and increased mortality rate	96 h	PS, 40 nm 0.5–45 mg/L, 0.5–10 mg/L	[173]
	*Ceriodaphnia silvestrii* and *Hyphessobrycon eques*	CuO-NPs	Decrease in reproduction, feeding inhibition, increased reactive oxygen species (ROS)	0, 24 and 48 h	HD, 30 nm, acute, 0, 0.7, 10.0, 13.0, 16.0, 19.0 µg/L, chronic, 0, 0.5, 1.0, 2.0, 4.0, 8.0, 10.0 µg/L	[174]
	*Clarias batrachus*	CuNPs	Testis-related genes showed upregulation, increase in level of androgens, and disruption in structural analysis	21 days	<50 nm, 100 µg/L	[175]
	*Danio rerio*	CuNPs	Retarded hatching embryos, causing morphological malformation and mortality in the gastrula stage	96 h	69 ± 18 nm, 0.01, 0.05, 0.1, 0.5, 1 mg/L	[179]
	*Leiarius pictus*	CuO-NPs	The developmental abnormalities caused by dissolution of internalized CuO-NPs	96 hpf	~10 nm, 20–2000 ppb commercial nano-CuO, 380–38,000 ppb synthesized nano-CuO	[180]
Heart	*Danio rerio*	CuO-NPs	Prevention of looping of the heart tube during cardiogenesis, also disturbing the dorsoventral patterning, increasing *wnt8* and *vent* expression	120 hpf	100–500 nm, 0.1, 0.5, 5, and 50 mg/L	[176]
Brain	*Tilapia mossambica*	CuNPs	Reduction in oxidative stress enzymes glutathione (GSH), glutathione-s-transferase (GST), and acetylcholine esterase (AChE)	6 days	15 mg/L	[167]
	*Danio rerio*	CuNPs	Reduced lateral line neuromasts (LLN) and performance of rheotaxis	96 h	20 ± 9 nm, 50 and 225 µg/L	[177]
	*Oncorhynchus mykiss*	CuNPs	Oxidative stress—increment in the ratio of oxidized to reduced glutathione	12 h	PS, <50 nm, 50 µg/L	[178]
	*Cyprinodon variegatus*	CuO-NPs	Behavioral changes, increase in mucus secretion, and loss in equilibrium	7 days	PS, 40 nm, 5 and 50 mg/L	[181]

HD: hydrodynamic diameter; PS: particle size; FW: fresh water; BW: brackish water, TBARS: thiobarbituric acid reactive substances; MDA: malondialdehyde; T-SOD: superoxide dismutase; GSH: glutathione; CAT: catalase.

**Table 3:** The significant outcomes of the toxicity of copper and copper nanoparticles to fish based on different tissue types.

Tissues	Copper Ions	Copper Nanoparticles
Brain	Disruption in behavioral, olfactory, and chemosensory impairments	Increase in oxidative stress, behavioural impairments
Liver	Liver is the major site for copper accumulation Cytolysis, necrosis, pyknosis, fibrosis, and induced oxidative stress	Increase in oxidative stress markers MDA, T-SOD, GSH, CAT & Caspase Necrosis, and pyknotic nuclei
Heart	Increase in AChE Abrupt changes in heart beats	Prevention of looping of the heart tube during cardiogenesis
Reproduction organs	Copper accumulation, interference with egg hatching and spermatogenesis, Increase in T3&T4 in ovary	Developmental abnormalities Retard hatching embryos Increase in ROS
Gill	Inhibition of Na^+^/K^+^ ATPase activity	Reduction in branchial Na^+^/K^+^ ATPase, Increased oxidative stress, Inhibition of AChE
Kidney	Damage to renal tubules, pronephros, high buffering capacity of copper	Tubular vacuolization and necrosis Glomerular shrinkage Macrophages aggregation

AChE: Acetylcholine esterase; CAT: Catalase; GSH: Glutathione; MDA: Malondialdehyde; ROS: Reactive oxygen species; T-SOD: Total superoxide dismutase.
